# Supplementary material for: Seminoma and Embryonal Carcinoma Footprints Identified by Analysis of Integrated Genome-Wide Epigenetic and Expression Profiles of Germ Cell Cancer Cell Lines
Source: PLoS One. 2014 Jun 2;9(6):e98330. doi: 10.1371/journal.pone.0098330 (PMC4041891; doi:10.1371/journal.pone.0098330)
Supplement: File S1 — ZIP file containing DMRforPairs output for significant regions. Please start from the html files. (ZIP) [file pone.0098330.s008.zip › figures/35507.pdf]

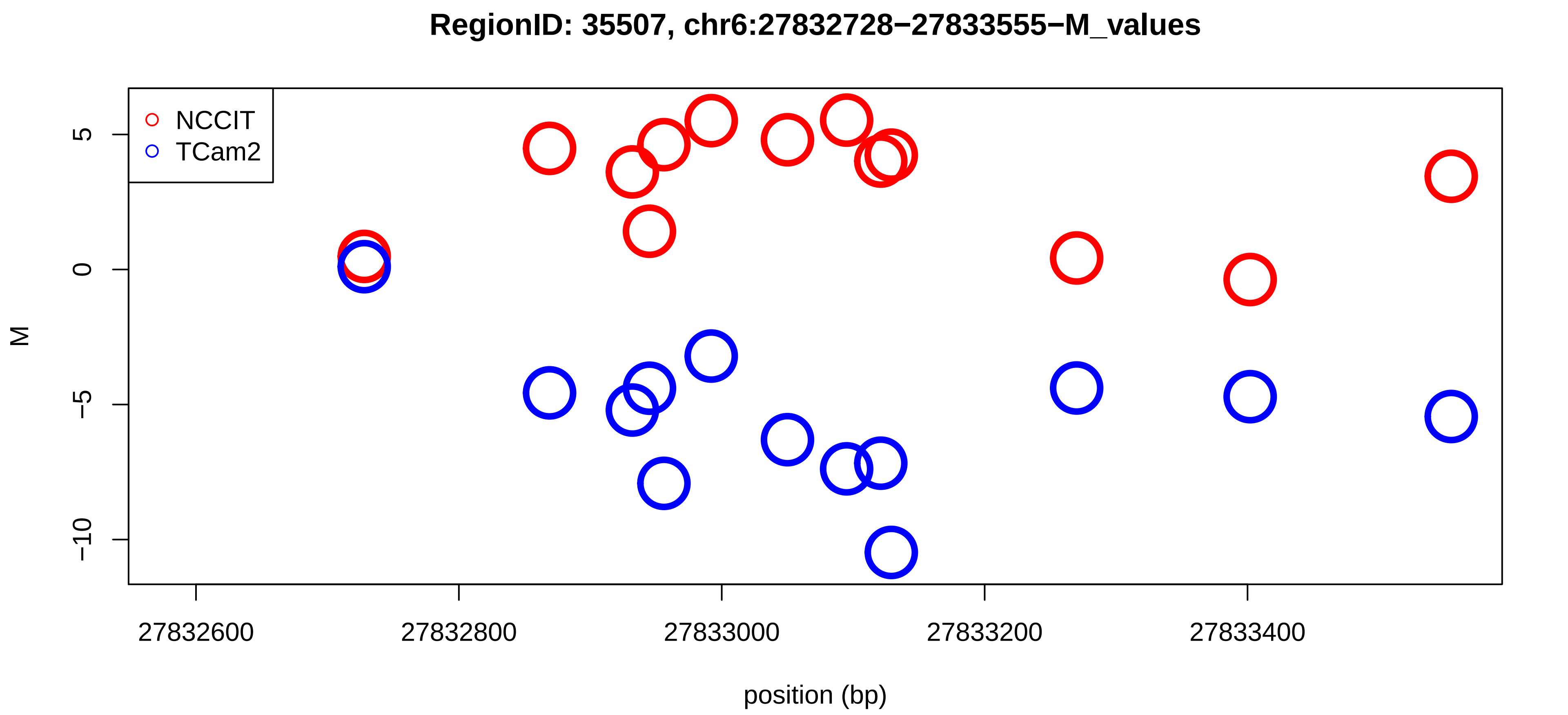
RegionID: 35507, chr6:27832728−27833555−M_values
NCCIT
TCam2
M
5
0
−5
−10
27832600
27832800
27833000
27833200
27833400
position (bp)

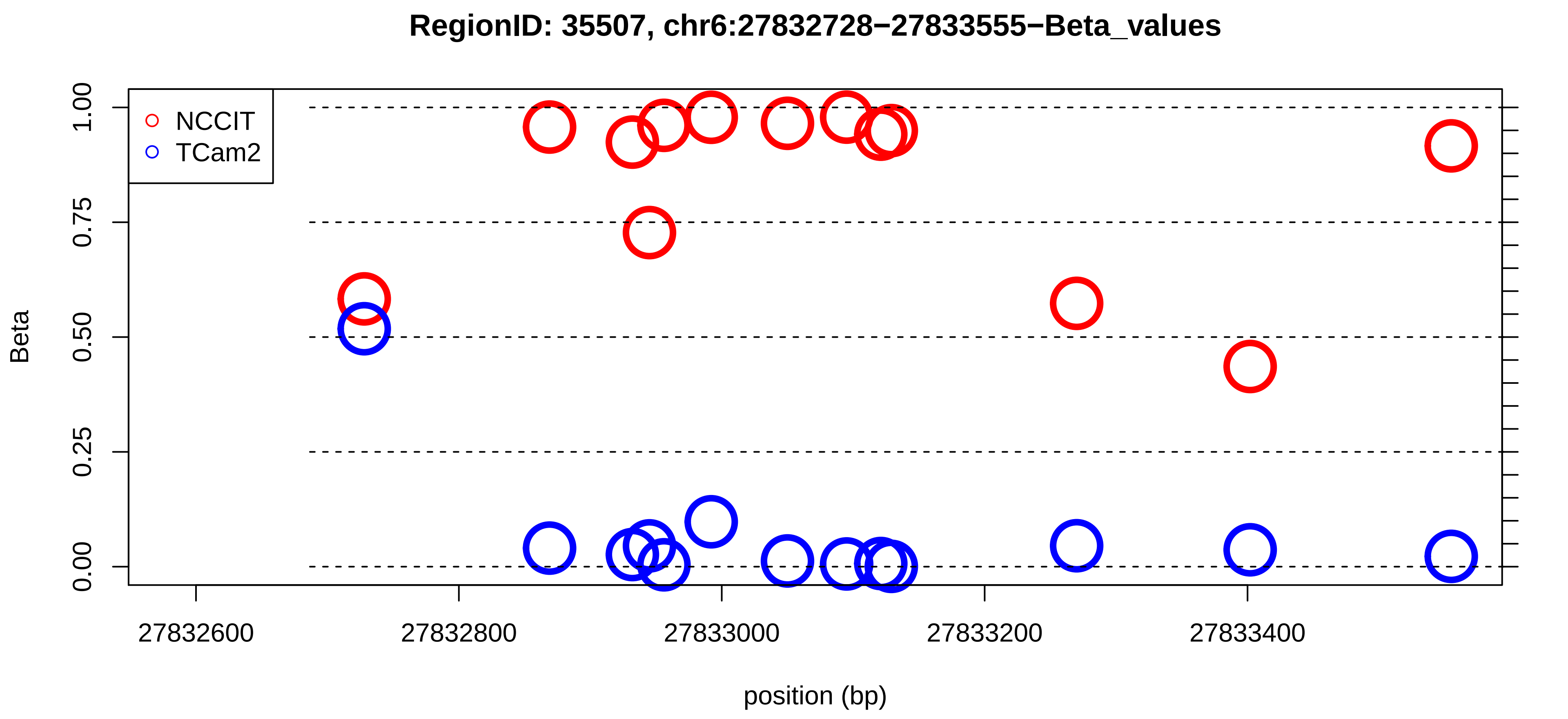
RegionID: 35507, chr6:27832728−27833555−Beta_values
NCCIT
TCam2
Beta
1.00
0.75
0.50
0.25
0.00
27832600
27832800
27833000
27833200
27833400
position (bp)

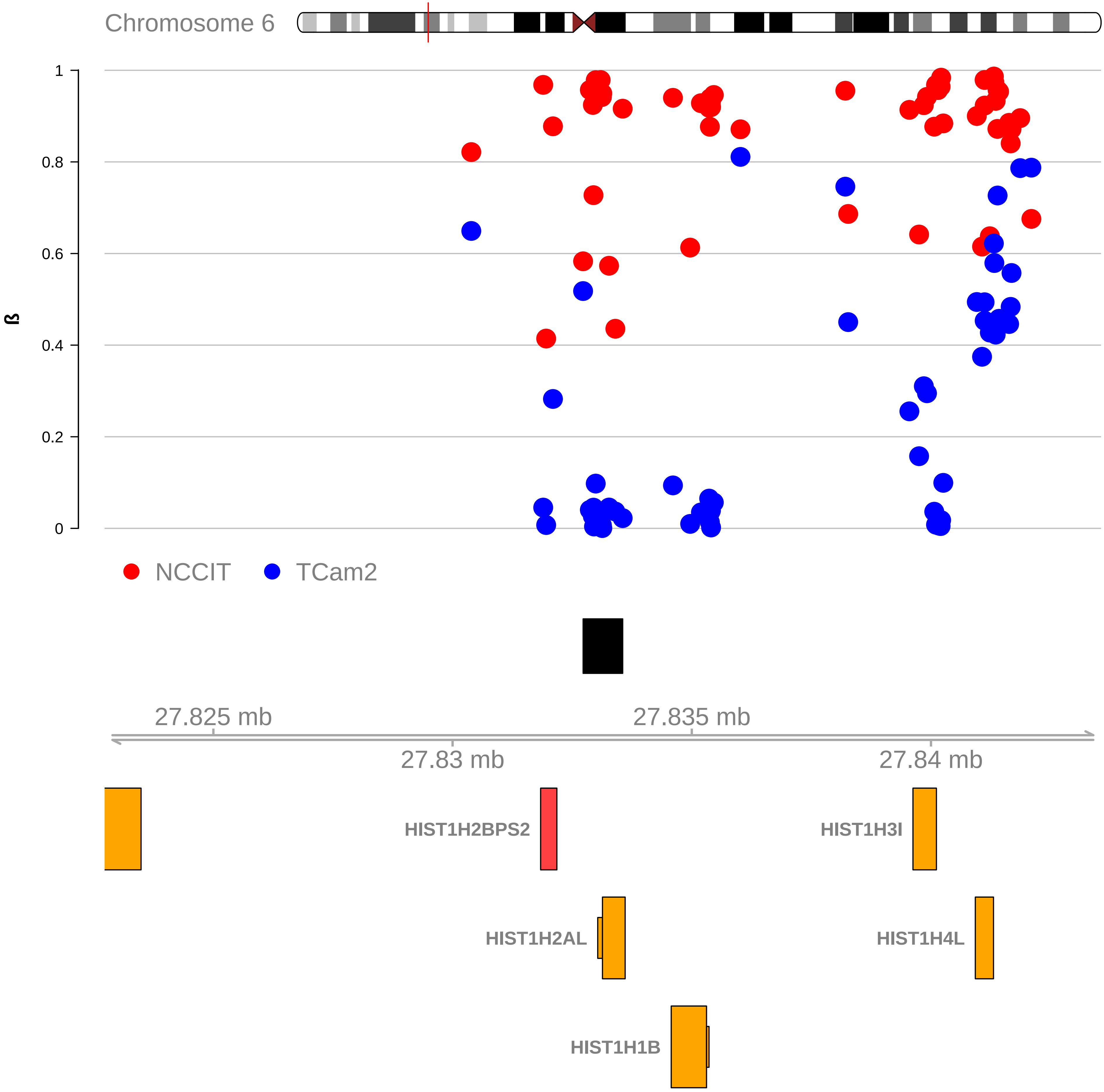

Chromosome 6
1
0.8
0.6
0.4
0.2
0
ß
NCCIT
TCam2
27.825 mb
27.835 mb
27.83 mb
27.84 mb
HIST1H2BPS2
HIST1H3I
HIST1H2AL
HIST1H4L
HIST1H1B
